# Risks and Probabilities of Adverse Pregnancy Outcomes in Patients Undergoing Trial of Labor after Cesarean—A Retrospective Study

**DOI:** 10.3390/diagnostics14161715

**Published:** 2024-08-07

**Authors:** Alexandru Carauleanu, Iustina Solomon-Condriuc, Petronela Vicoveanu, Demetra Socolov, Ioana-Sadiye Scripcariu, Ingrid-Andrada Vasilache, Iulian-Valentin Munteanu, Luiza-Maria Baean, Ana-Maria Adam, Raluca Mogos, Liliana Gheorghe

**Affiliations:** 1 Department of Mother and Child Care, “Grigore T. Popa” University of Medicine and Pharmacy, 700115 Iasi, Romania; ale.carauleanu@umfiasi.ro (A.C.); demetra.socolov@umfiasi.ro (D.S.); ioana.scripcariu@umfiasi.ro (I.-S.S.); ingrid-andrada.vasilache@umfiasi.ro (I.-A.V.); raluca-anamaria-i-mogos@d.umfiasi.ro (R.M.); 2 Department of Mother and Newborn Care, Faculty of Medicine and Biological Sciences, ‘Ștefan cel Mare’ University, 720229 Suceava, Romania; 3 Clinical and Surgical Department, Faculty of Medicine and Pharmacy, ‘Dunarea de Jos’ University, 800216 Galati, Romania; valentin.munteanu@ugal.ro (I.-V.M.), ana-maria.adam@ugal.ro (A.-M.A.); 4 Surgical Department, Faculty of Medicine, “Grigore T. Popa” University of Medicine and Pharmacy, 700115 Iasi, Romania; maria-luiza.cobzeanu@umfiasi.ro (L.-M.B.); liliana.gheorghe@umfiasi.ro (L.G.)

**Keywords:** trial of labor after cesarean, perinatal risk, prediction, perinatal complications

## Abstract

(1) Background: Trial of labor after cesarean (TOLAC) can be associated with significant maternal and neonatal complications, and the aim of this retrospective study was to calculate the risks and probabilities of these complications in two tertiary maternity centers in Romania. (2) Methods: A total of 216 patients who attempted TOLAC were included in the study and were segregated into two groups, depending on TOLAC success. Medical records were assessed, and clinical data were used to determine the maternal and neonatal risks and complications, using multinomial logistic regression and postestimation predictions. (3) Results: Our data indicated that patients who had a failed TOLAC had significantly higher risks and probabilities of uterine rupture, either complete or incomplete; intensive care unit (ICU) admission; and emergency hysterectomy. The newborns of these mothers had significantly higher risks and probabilities of low Apgar score at birth, neonatal intensive care unit (NICU) admission, and invasive ventilation. (4) Conclusions: Failed TOLAC could lead to significant maternal and neonatal complications, and women who attempt this procedure should be monitored in a tertiary center where a multidisciplinary team and an emergency operating room are available.

## 1. Introduction

The last few decades of obstetrical practice have been marked by increasing cesarean delivery rates worldwide. The Eurostat data indicate that, in Romania, 447.78 cesarean sections were performed per 100,000 inhabitants in 2021, ranking third in Europe for this metric after Türkiye (749.73 cesarean deliveries per 100,000 inhabitants) and Cyprus (686.84 cesarean deliveries per 100,000 inhabitants) [1]. Years ago, obstetricians adhered to the dictum “once a cesarean, always a cesarean” [2]. However, recent clinical data support alternatives to elective repeat cesarean delivery (ERCD), such as a trial of labor after cesarean (TOLAC), which can lead to a successful vaginal birth after cesarean (VBAC).

A successful approach to TOLAC could significantly reduce the high cesarean rates as well as the financial burden on the healthcare system. However, the acceptability rates of this procedure vary greatly among different countries. For example, the reported TOLAC rates are approximately 74.9% in Norway [3], 52% in Finland [4], and 21.7% in the United States of America [5].

The TOLAC’s acceptability depends on various factors, such as the obstetricians’ experience with and attitude towards this procedure, the patients’ wish to experience a vaginal birth after cesarean, organizational support, prompt access to an operating room, and a multidisciplinary approach [6,7,8]. Thus, it is necessary to take into consideration all of these factors when evaluating the TOLAC rates in various healthcare systems.

The complication rates after TOLAC or ERCD could serve as an orientation for choosing the most suitable modality of birth. To date, there has been no randomized controlled trial in the literature that compares the adverse neonatal and maternal outcomes in patients undergoing TOLAC or ERCD, mainly due to ethical concerns. Thus, literature regarding this topic is comprised of observational studies. The ACOG practice bulletin published in 2019 regarding the VBAC outlined a higher risk of infectious morbidity and uterine rupture in patients undergoing TOLAC versus those who had an ERCD [9]. Moreover, the newborns had a higher risk of respiratory morbidity and neonatal intensive care unit admission, as well as higher neonatal morbidity rates.

A retrospective cohort study conducted by Rudzinski et al. evaluated the maternal and neonatal outcomes in patients undergoing VBAC or ERCD [10]. Its results indicated a higher risk of preterm birth or post-term birth, premature rupture of membranes, intrahepatic cholestasis of pregnancy, postpartum anemia, and peripartum blood loss >1 L in patients who achieved a VBAC, compared with those who underwent ERCD.

By far, the most important complication that can occur during TOLAC is uterine rupture, and its incidence is approximately 0.1–3.9% [11,12,13,14], depending on the study population and number of previous cesarean deliveries. Its management requires a multidisciplinary team and prompt access to an operating room, thus rendering it a necessity to give birth within a tertiary center, especially in the cases of patients with high-risk pregnancies.

Statistics about maternal and neonatal complication rates in patients who underwent TOLAC in Romania are lacking. Thus, the aim of this retrospective study was to determine the risks and probabilities of maternal and neonatal adverse outcomes in a cohort of patients with failed TOLAC from two tertiary maternity centers in Romania.

## 2. Materials and Methods

This observational retrospective study included 216 pregnant patients who attempted TOLAC at two tertiary maternity centers in Romania between January 2007 and December 2023. Ethical approval for this study was obtained from the Institutional Ethics Committees of “Cuza voda” Clinical Hospital of Obstetrics and Gynecology in Iasi (9836/9 September 2020) and “Saint John” Emergency Hospital in Suceava (7/21 January 2022). All patients signed informed consent forms for the medical data processing.

In this study, we included pregnant patients with singleton pregnancies, a personal history of a low-transverse cesarean section, a cephalic presentation, and an absence of contraindications for VBAC, who underwent a TOLAC. The exclusion criteria comprised multifetal gestation, a history of major uterine surgical procedures, previous inverted T or J incisions, low vertical uterine incisions or significant inadvertent uterine extension at the time of primary cesarean, placenta accreta spectrum disorder, and a previous history of uterine rupture, stillbirths, or other complications that generally constitute contraindications for vaginal birth, as well as incomplete medical records.

A total of 216 patients who attempted TOLAC were segregated into two groups considering the success of this procedure: group 1 (successful TOLAC followed by VBAC, *n* = 182 patients) and group 2 (failed TOLAC followed by cesarean section, *n* = 34 patients).

Data from their medical records were assessed, and the following variables were taken into consideration for further analysis: demographic data, parity, personal history of cesarean delivery, type of uterine incision, previous vaginal births and VBACs, the type of labor (induced/spontaneous), and a personal history of adverse pregnancy outcomes or maternal disorders, as well as maternal and neonatal complications.

A complete uterine rupture was defined as a complete disruption of all uterine layers, including the serosa, leading to discernable changes in maternal or fetal status. Partial uterine rupture, also known in the literature as dehiscence, was defined as an incomplete disruption of the uterine layers that is not readily discernible by signs or symptoms. The complete or partial uterine rupture was determined using intrapartum ultrasound performed during labor or at the time of surgical intervention.

The statistical analysis comprised, in the first place, a univariate analysis that used Pearson’s χ^2^ test for categorical variable comparison and Student’s *t*-test for continuous variable comparison.

This was followed by multinomial logistic regression, which allowed the calculation of relative risk ratios (RRR) and 95% confidence intervals (CI) for adverse maternal and neonatal outcomes in cases of failed TOLAC. We also calculated the probability of adverse maternal and neonatal outcomes in cases of failed TOLAC using postestimation margins and predictions. The results were graphically represented using scatterplots.

Lastly, we used the Mantel–Haenszel test to evaluate the risk of adverse pregnancy outcomes for the failed TOLAC group in comparison with the successful TOLAC group and reported the results as a crude and adjusted odds ratio with a 95% confidence interval (CI). The magnitude of the confounding was calculated using the following formula: (crude OR- adjusted OR)/adjusted OR.

The following confounding variables were taken into consideration as to adverse maternal outcomes: maternal age; obesity; parity; previous cesarean, vaginal, or VBAC deliveries; induction of labor; birthweight; and gestational age at birth. We also controlled for the last two confounders (birthweight and gestational age at birth) when we evaluated the odds of adverse neonatal outcomes.

A *p*-value of less than 0.05 was considered statistically significant. These analyses were conducted using STATA SE (version 18.5, 2024, StataCorp LLC, College Station, TX, USA).

## 3. Results

A total of 216 patients with singleton pregnancies and a previous history of cesarean section who attempted a TOLAC were included in the study, and their clinical characteristics are presented in Table 1. The TOLAC success rate for the evaluated cohort was 84.25%. Our results indicated that the evaluated groups were relatively similar in terms of age (*p* = 0.48) and living environment (*p* = 0.31).

The mean parity (and standard deviation) of the patients who achieved a successful TOLAC was significantly higher compared to that of patients who had a failed procedure and underwent ERCD (2.90 ± 1.20 versus 2.26 ± 0.56, *p* = 0.001). This is a measure of how many deliveries of viable fetuses a patient has had in her obstetrical history.

Also, the mean number (and standard deviation) of previous vaginal deliveries was significantly higher for the first group of patients, compared with the second group (0.82 ± 1.18 versus 0.23 ± 0.49, *p* = 0.002). Although it was not statistically significant, five patients (2.74%) who achieved a successful TOLAC had achieved a previous VBAC, as compared to none in the second group. We did not find any statistically significant difference between groups regarding the gestational age at birth (36.28 ± 4.14 versus 36.5 ± 4.04) and birthweight (2645.60 ± 821.68 versus 2714.70 ± 817.92).

Finally, the majority of patients included in the first group had spontaneous labor, while in the second group, the majority of the patients had induced labor using oxytocin or mechanical means (*p* = 0.014). No statistically significant differences were noted regarding the personal history of gestational diabetes (*p* = 0.39) or preeclampsia/gestational hypertension (*p* = 0.91).

We have employed a multinomial logistic regression analysis for the identification of the RRR and their 95%CI for maternal and neonatal complications in cases of failed TOLAC, and the results are presented in Table 2. Our data indicated that patients who had a failed TOLAC had a significantly higher risk of uterine rupture, either complete or incomplete (RRR: 4.68, 95%CI: 1.81–12.07, *p* = 0.001); intensive care unit (ICU) admission (RRR: 1.79, 95%CI: 0.70–4.58, *p* = 0.03); and emergency hysterectomy (RRR: 19.28, 95%CI: 3.70–100.32, *p* < 0.001).

Also, the newborns of these mothers had a significantly higher risk of low Apgar score at birth (RRR: 2.94, 95%CI: 1.35–6.38, *p* = 0.006), neonatal intensive care unit (NICU) admission (RRR: 5.10, 95%CI: 2.32–11.21, *p* < 0.001), and invasive ventilation (RRR: 4.11, 95%CI: 1.360–12.46, *p* = 0.012).

Finally, we calculated the probabilities of maternal and neonatal complications depending on the TOLAC success, and the results are presented in Table 3. The probability of uterine rupture, either complete or incomplete, was 26% in cases of failed TOLAC and 7% in cases of successful TOLAC, and the difference was statistically significant (*p* < 0.001).

Also, the probabilities of ICU admission (20 versus 12%, *p* = 0.003) and emergency hysterectomy (17 versus 1%, *p* < 0.001) were significantly higher in cases of failed TOLAC compared to those determined in cases of successful TOLAC.

Regarding neonatal complications, our results indicated significantly higher probabilities of low Apgar scores at birth (41 versus 19%, *p* < 0.001), NICU admission (47 versus 14%, *p* < 0.001), and invasive ventilation (17 versus 4%, *p* = 0.002).

**Figure 1:**
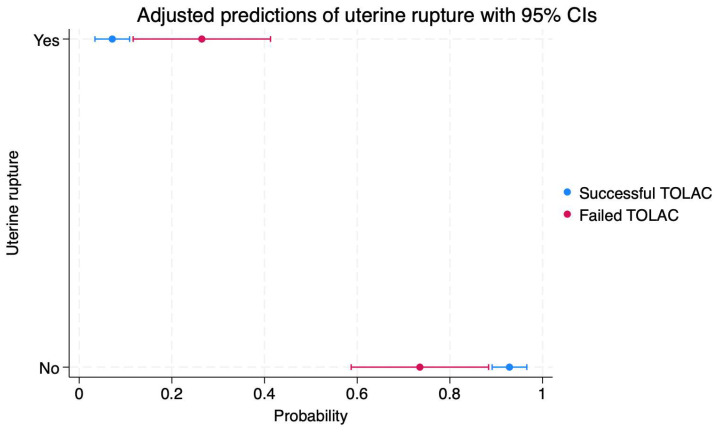
Scatterplot indicating the probabilities of uterine rupture in cases of failed or successful TOLAC. Legend: TOLAC—trial of labor after cesarean; CI—confidence interval.

**Figure 2:**
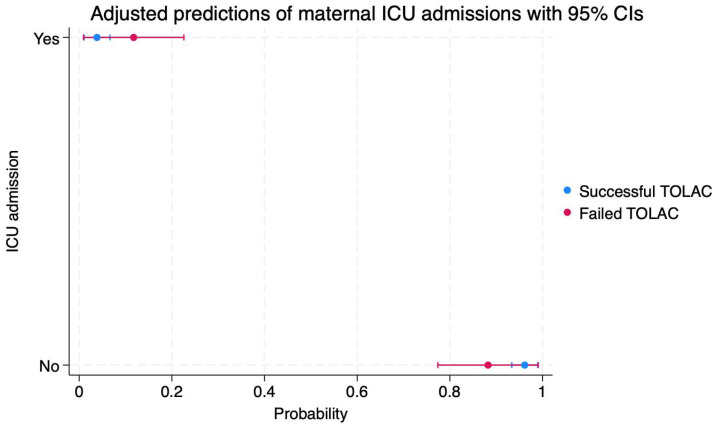
Scatterplot indicating the probabilities of maternal ICU admission in cases of failed or successful TOLAC. Legend: TOLAC—trial of labor after cesarean; ICU—intensive care unit; CI—confidence interval.

**Figure 3:**
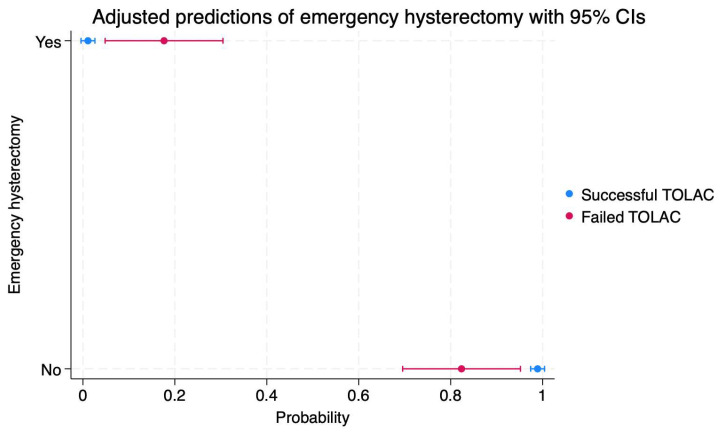
Scatterplot indicating the probabilities of emergency hysterectomy in cases of failed or successful TOLAC. Legend: TOLAC—trial of labor after cesarean; CI—confidence interval.

**Figure 4:**
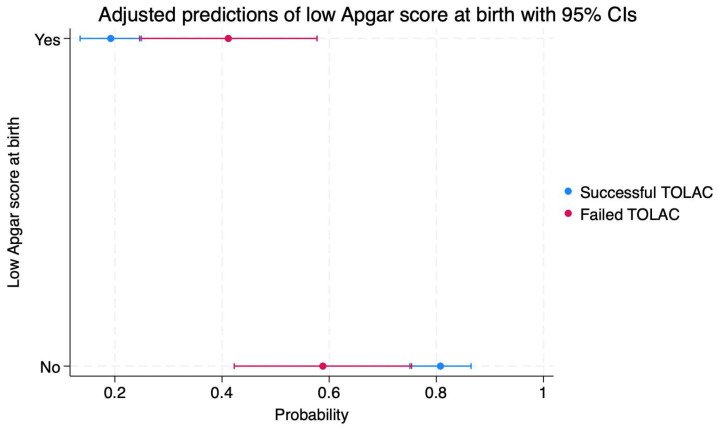
Scatterplot indicating the probabilities of a low Apgar score at birth in cases of failed or successful TOLAC. Legend: TOLAC—trial of labor after cesarean; CI—confidence interval.

**Figure 5:**
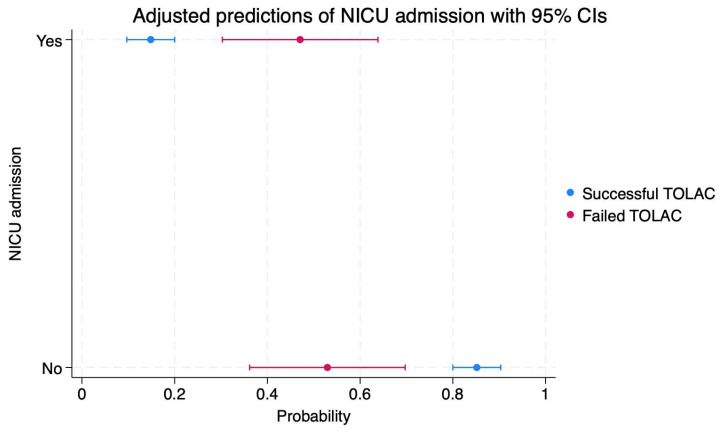
Scatterplot indicating the probabilities of NICU admission in cases of failed or successful TOLAC. Legend: TOLAC—trial of labor after cesarean; NICU—neonatal intensive care unit; CI—confidence interval.

**Figure 6:**
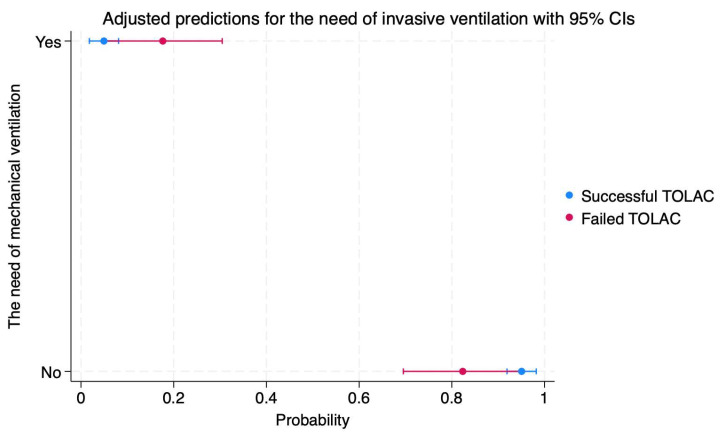
Scatterplot indicating the probabilities for the need of invasive ventilation in cases of failed or successful TOLAC. Legend: TOLAC—trial of labor after cesarean; CI—confidence interval.

We calculated the probabilities of maternal and neonatal complications depending on TOLAC success, and the results are presented in Figure 1, Figure 2, Figure 3, Figure 4, Figure 5 and Figure 6. The first figure includes a scatterplot representation indicating the probability of uterine rupture, depending on the success of TOLAC.

Thus, the probability of a lack of uterine rupture in a case of successful TOLAC is 92% (95%CI: 0.89–0.96), and in a case of a failed TOLAC, it is 73% (95%CI: 0.58–0.88). On the other hand, the probability of uterine rupture in cases of a successful TOLAC is 7% (95%CI: 0.03–0.10), and in cases of a failed TOLAC, it is 26% (95%CI: 0.11–0.41).

Patients who achieved a successful TOLAC and VBAC had a 96% probability of not being admitted to the ICU (95%CI: 0.93–0.98), as well as a 3% probability of being admitted to this ward (95%CI: 0.01–0.06) (Figure 2).

Conversely, patients with a failed TOLAC had an 88% probability of not being admitted to the ICU (95%CI: 0.77–0.99), as well as an 11% probability of being admitted to this ward (95%CI: 0.01–0.22) (Figure 2).

The scatterplot in Figure 3 indicates a 17% probability of emergency hysterectomy (95%CI: 0.04–0.30), as well as a probability of 82% of not needing this procedure (95%CI: 0.69–0.95), in patients with failed TOLAC.

On the other hand, the same scatterplot outlines a probability of 1% for emergency hysterectomy (95%CI: −0.04–0.02), as well as a probability of 98% of not needing this procedure (95%CI: 0.97–1), in patients with successful TOLAC.

The probability of a low Apgar score at birth in the case of successful TOLAC is 19% (95%CI: 0.13–0.24), and in the case of failed TOLAC, it is 41% (95%CI: 0.24–0.57) (Figure 4). On the other hand, the probability of an Apgar score at birth being greater than seven in the case of a successful TOLAC is 80% (95%CI: 0.75–0.86), and in the case of a failed TOLAC, it is 58% (95%CI: 0.42–0.75).

Newborns from mothers who achieved a successful TOLAC and VBAC had an 85% probability of not being admitted to the NICU (95%CI: 0.80–0.90) as well as a 14% probability of being admitted to this ward (95%CI: 0.09–0.19) (Figure 5).

Conversely, newborns from mothers with a failed TOLAC had a 52% probability of not being admitted to the NICU (95%CI: 0.36–0.69) as well as a 47% probability of being admitted to this ward (95%CI: 0.30–0.63) (Figure 5).

Newborns from mothers who achieved a successful TOLAC and VBAC had a 95% probability of not needing invasive ventilation (95%CI: 0.91–0.98) as well as a 4% probability of needing this procedure (95%CI: 0.01–0.08) (Figure 6).

Conversely, newborns from mothers with a failed TOLAC had an 82% probability of not needing invasive ventilation (95%CI: 0.69–0.95), as well as a 17% probability of needing this procedure (95%CI: 0.04–0.30) (Figure 6).

During the subsequent stage of our research, we assessed the odds of the most important adverse maternal outcomes for the failed TOLAC group in contrast to the successful TOLAC group, considering also the influence of confounding variables such as maternal age; obesity; parity; previous cesarean, vaginal, or VBAC deliveries; induced labor; birthweight; and gestational age at birth. The findings are displayed in Table 4.

Our results indicated significantly higher odds of uterine rupture, considering the crude estimates (OR: 4.68, 95%CI: 1.76–12.43, *p* = 0.006), the adjusted estimates using all confounders (OR: 4.85, 95%CI: 0.95–24.59), and the adjusted estimates based on each separate confounding variable (*p* < 0.05).

In order for a confounding effect to be significant, there should be at least a 10% difference in the odds. Thus, the induction of labor had the most significant confounding effect on uterine rupture in our cohort of patients (12%).

When we evaluated the odds of emergency hysterectomy, our data indicated that these odds were significantly higher for the failed TOLAC group, considering the crude estimates (OR: 19.28, 95%CI: 3.37–110.3, *p* < 0.001), the adjusted estimates for all confounding variables (OR: 12, 95%CI: 0.63–126.97, *p* = 0.03), and the adjusted estimates based on each separate confounding variable (*p* < 0.001).

The magnitude of the confounding effect on the evaluated outcomes was significant for the previous number of cesarean (22%) or vaginal deliveries (47%), as well as for the induction of labor (23%). The overall effect of all confounding variables was 61%.

Finally, the odds of maternal ICU admission remained significantly higher for the failed TOLAC group, considering the crude estimates (OR: 4.31, 95%CI: 1.25–14.81, *p* = 0.01), the adjusted estimates for all confounding variables (OR: 12, 95%CI: 0.68–95.39, *p* = 0.01), and the adjusted estimates based on each separate confounding (*p* < 0.05). The magnitude of the confounding effect on the evaluated outcomes was significant for obesity (11%), parity (14%), and induction of labor (21%). The overall effect of all confounding variables was 64%.

Finally, we assessed the odds of the most important adverse neonatal outcomes for the failed TOLAC group in contrast to the successful TOLAC group, considering also the influence of confounding variables such as birthweight and gestational age at birth. The findings are displayed in Table 5.

Our results indicated significantly higher odds of a low Apgar score at birth, considering the crude estimates (OR: 2.29, 95%CI: 1.02–5.11, *p* = 0.03), the adjusted estimates using all confounders (OR: 6.48, 95%CI: 1.77–23.61, *p* = 0.001), and the adjusted estimates based on each separate confounding (*p* < 0.05). Both birthweight (40%) and gestational age at birth (67%) had a significant confounding effect for the evaluated outcomes. The overall confounding effect was 65%.

When we evaluated the odds of NICU admission, our data indicated that they were significantly higher for the failed TOLAC group, considering the crude estimates (OR: 5.10, 95%CI: 2.23–11.65, *p* < 0.001), the adjusted estimates for all confounding variables (OR: 18, 95%CI; 3.68–113.52, *p* < 0.001), and the adjusted estimates based on each separate confounding (*p* < 0.001). The magnitude of the confounding effect on the evaluated outcome was significant for birthweight (69%) and gestational age at birth (61%). The overall confounding effect of these variables was 72%.

The odds of invasive ventilation remained significantly higher for the failed TOLAC group, considering the crude estimates (OR: 3.31, 95%CI: 1.02–10.75, *p* = 0.03), the adjusted estimates for all confounding variables (OR: 4.98, 95%CI: 1.34–18.44, *p* = 0.007), and the adjusted estimates based on each separate confounding (*p* = 0.01). The magnitude of the confounding effect on the evaluated outcomes was significant for birthweight (22%) and gestational age at birth (18%). The overall effect of all confounding variables was 34%.

## 4. Discussion

VBAC could be a solution useful for reducing the rates of cesarean sections, especially in countries where these rates exceed 50% of total deliveries. It is known that the majority of cesarean sections performed are ERCD, and the risk of the occurrence of complications, especially placenta accreta spectrum disorder, is proportional to the number of uterine scars [15,16].

When planning the mode of birth, it is important to communicate the risks and benefits of each type of delivery to expectant mothers. The maternal and neonatal complications cited in the literature have variable incidence rates depending on the studied population and type of medical center, and these aspects outline the need for local observational studies that will better reflect the risks associated with each procedure. Thus, in this observational retrospective study, we aimed to offer our insights regarding the risks and probabilities of adverse maternal and neonatal outcomes in women who attempted TOLAC in two tertiary centers in Romania.

Our results pointed out higher risks and probabilities of uterine rupture, either complete or incomplete; ICU admission; and emergency hysterectomy for mothers with failed TOLAC. Also, the newborns of these mothers had a significantly higher risk of a low Apgar score at birth, NICU admission, and invasive ventilation. Moreover, the risk of important adverse maternal and neonatal outcomes remained significantly higher for the failed TOLAC group even after adjusting for confounders. These results are in line with recent published data.

For example, He et al. conducted a retrospective study that included 720 pregnant patients with a personal history of cesarean section and investigated the risk factors as well as the outcomes for failed TOLAC [17]. In that study, the success rate of TOLAC was 84.2%, and this result is similar to the one obtained in our cohort of patients—84.25%.

At the same time, these authors indicated a significantly higher risk of intrapartum hemorrhages, as well as significantly higher rates of NICU admission in the failed TOLAC group [17]. The most important risk factor for TOLAC failure and uterine rupture was the induction of labor. In our study, patients who had a successful TOLAC and, implicitly, VBAC, had significantly higher rates of spontaneous labor.

Qiu et al. conducted a systematic review and meta-analysis that evaluated the safety of TOLAC versus ERCD in patients with a prior cesarean delivery [18]. For the quantitative analysis of this study, the authors included data gathered from 13 studies and 676,532 pregnant patients. Their results indicated a significant risk of uterine rupture in patients attempting TOLAC compared with those who choose ERCD (odds ratio, OR = 3.35, 95%CI: 1.57–7.15, *I*^2^ = 81%) [18]. Also, increased risks of perinatal asphyxia (OR = 2.32, 95%CI: 1.76–3.08, *I*^2^ = 0%) and perinatal death (OR = 2.32, 95%CI: 1.76–3.08, *I*^2^ = 0%) were calculated for newborns from mothers who attempted TOLAC.

On the other hand, the meta-analysis did not find a significantly higher risk of blood transfusions (OR = 1.24, 95%CI: 0.72–2.12], *I*^2^ = 95%) or puerperal infection (OR = 1.11, 95%CI: 0.77–1.60, *I*^2^ = 95%) in TOLAC patients, compared to ERCD patients [18]. Both of these results are characterized by marked heterogeneity.

In our study, the necessity of blood transfusions did not significantly differ between groups (RRR: 1.30, 95%CI: 0.05–3.16, *p* = 0.992), and the probabilities of this procedure were 4% in the case of failed TOLAC (followed by ERCD) and 2% in the case of successful TOLAC (followed by VBAC) (*p* = 0.48).

Another recent meta-analysis aimed to comparatively analyze the rates of maternal and neonatal complications in TOLAC versus ERCD patients [19]. This meta-analysis included 13 studies and demonstrated a significantly higher risk of uterine rupture (OR = 2.01, 95%CI: 1.48–2.74, *p* < 0.00001) and low Apgar score at birth (OR = 2.17, 95%CI: 1.69–2.77, *p* < 0.00001) in TOLAC deliveries, compared to ERCD. However, the authors did not find a significant difference between groups regarding the risk of NICU admission, postpartum infection or hemorrhage, or maternal blood transfusions, or a significant difference in hysterectomy rates.

Uterine rupture is a life-threatening complication, and current guidelines recommend conducting TOLAC in facilities that are able to perform an emergency cesarean birth [9,20]. By no means should pregnant patients attempt a TOLAC at home or in a birthing center where an emergency operating room is unavailable. There are several risk factors for uterine rupture, such as labor induction (especially with prostaglandins), a uterine body scar, a previous history of uterine rupture, or a fetal birthweight greater than 4500 g [11,12]. There are insufficient data to contraindicate TOLAC in patients with two uterine scars due to cesarean deliveries, and this procedure should be conducted with prudence in this category of patients [20]. Our results confirmed the influence of labor induction as a confounder with significant effects on the probability of the occurrence of uterine rupture, emergency hysterectomy, and maternal ICU admission.

All guidelines support the use of epidural analgesia for patients undergoing TOLAC, and current evidence does not indicate a reduction in the success rate of TOLAC when using this procedure [9,20]. However, none of our patients benefited from this procedure during labor, due to logistical reasons.

The prediction of TOLAC failure has gained popularity in recent years, and it has been a subject of controversy. Several models have been proposed, and many used clinical and ultrasound parameters which were analyzed using logistic regression or machine learning-based algorithms. For example, Baranov et al. conducted a retrospective study that aimed to validate a prediction model for VBAC in a cohort of 630 pregnant patients with singleton pregnancies at term and fetuses in cephalic presentation [21]. This model was first proposed by Grobman et al. [22], and included the following predictors: maternal age, body mass index, race, any prior vaginal delivery, prior VBAC, and recurring indications for prior cesarean delivery. In the validation phase, this model had an area-under-the-curve value of 0.70 (95%CI: 0.66–0.74).

The initial model proposed by Grobman et al. has been modified, and it now comprises two forms: one that is administered in the early antenatal visit (maternal age, BMI, pre-pregnancy weight, obstetric history, arrest disorder indication for prior cesarean, and treated chronic hypertension), as well as a form that is administered at the labor ward admission (maternal age, BMI, weight at delivery, obstetric history, arrest disorder indication for prior cesarean, gestational age at admission, hypertensive disorder of pregnancy, cervical dilatation and effacement, and fetal station) [23,24]. It is also known as the Maternal–Fetal Medicine Units (MFMU) VBAC calculator.

Meyer et al. investigated the predictive performance of three machine learning-based algorithms: Random Forest (RF), regularized regression (GLM), and eXtreme gradient-boosted decision trees (XGBoost) as to the prediction of TOLAC success, and compared their performance to that of the MFMU model [25]. The authors used a cohort of 989 pregnant patients with singleton pregnancies from a tertiary maternity center, and included their clinical characteristics in further analyses.

Their results indicated that the TOLAC success rate was 85.6%, which is comparable to that found in our study. The predictive performance of the evaluated models was modest, with AUC values of 0.351 for RF, 0.350 for XGBoost, 0.336 for GLM, and 0.325 for the MFMU-C model [25]. Height and prior vaginal delivery were two of the three most significant predictors of TOLAC success in all ML models. Maternal height was more accurate than maternal weight, and prior arrest of descent was more accurate than prior arrest of dilatation.

Moreover, Yang et al. evaluated the predictive performance of five machine learning-based models (Random Forest, XGBoost, LightGBM, Decision Tree, and CatBoost) with that of a regression model for the prediction of VBAC success in a retrospective study [26]. These models were integrated into a decision-aid birth choice platform and included the following variables: maternal age, gravidity, body mass index, previous vaginal birth, prolonged labor, gestational diabetes, and chronic hypertension. With an accuracy of 0.91, the CatBoost model proved to be the most accurate during the trial phase. Alternatively, the models of Logistic Regression, Random Forest, XGBoost, LightGBM, and Decision Tree obtained relatively low accuracy values, with respective values of 0.69, 0.88, 0.82, 0.90, and 0.83.

Another study, conducted by Awawdeh et al., tested the predictive performance of four machine learning models (Decision Tree, Random Forest, K-Nearest Neighbors—KNN, and Logistic Regression) for the prediction of the individualized likelihood of mode of delivery in patients with a previous CD [27]. Their results indicated that the best model for the prediction was KNN, and it achieved an area-under-the-curve value of 0.812. These data suggest the possibility of using machine-learning based algorithms for making individualized decisions about the mode of birth in this category of patients.

This observational study has some particularities and limitations. First of all, its retrospective design did not allow us to evaluate the uterine scar thickness determined by ultrasound examination; data in the current literature support the use of this parameter as a predictor of uterine rupture [28,29].

Second, the TOLAC rates in our geographical regions are low, even though the TOLAC success rates are comparable to those published in the literature. Moreover, the acceptability of this procedure among Romanian patients has increased in recent years, and this constitutes a positive signal for an honest patient–obstetrician conversation regarding the real possibilities of VBAC in our country. Thus, there is a need to support a national health care strategy that promotes VBAC in selected patients, and this could ultimately reduce ERCD rates.

Third, the strong points of this study include the extended time-frame and the use of data gathered from two tertiary maternity centers that are representative of the region and benefit from multidisciplinary and experienced teams, modern operating rooms, and functioning NICU wards.

In this study, we comparatively evaluated only the clinical parameters of the included pregnant patients. We are conducting a prospective study in both tertiary units that incorporates clinical and ultrasound parameters for the prediction of TOLAC success. Thus, further studies should also include ultrasound parameters such as angle of progression, head–perineum and head–symphysis distance, fetal station, midline angle, and uterine scar thickness for better evaluation of labor dynamics.

The integration of a large number of clinical and ultrasound parameters could also enhance the predictive performance of various machine learning-based models, and this is another future direction of research. However, these models need to pass the test of external validation and should be tested in various clinical settings.

## 5. Conclusions

According to our data, patients with a failed TOLAC had a significantly higher risk and probability of admission to the ICU, emergency hysterectomy, and uterine rupture.

Additionally, the newborns of these mothers were at a considerably increased risk and probability of admission to the NICU, having low Apgar scores at birth, and needing invasive ventilation.

Further studies should incorporate large sets of clinical and ultrasound data into prediction models of TOLAC success, which should be further validated in prospective studies.

## Figures and Tables

**Table 1:** Clinical characteristics of included patients.

Characteristics	Group 1 (Successful TOLAC, *n* = 182 Patients)	Group 2 (Failed TOLAC, *n* = 34 Patients)	*p* Value
Age, years (mean and SD)	29.73 ± 6.24	30.52 ± 5.64	0.48
Medium (*n*/%)	Urban = 74 (40.66%) Rural = 108 (59.34%)	Urban = 15 (44.12%) Rural = 19 (55.88%)	0.31
Parity (mean and SD)	2.90 ± 1.20	2.26 ± 0.56	0.001
Number of previous CD (mean and SD)	1.07 ± 0.26	1.02 ± 0.17	0.15
Number of previous VD (mean and SD)	0.82 ± 1.18	0.23 ± 0.49	0.002
History of VBAC (*n*/%)	5 (2.74%)	0 (%)	0.32
Spontaneous labor (*n*/%)	143 (78.57%)	14 (41.18%)	0.014
History of gestational diabetes (*n*/%)	2 (1.10%)	1 (2.94%)	0.39
History of PE/GHTN (*n*/%)	6 (3.30%)	1 (2.94%)	0.91
Gestational age at birth, weeks (mean and SD)	36.28 ± 4.14	36.5 ± 4.04	0.78
Birthweight, g (mean and SD)	2645.60 ± 821.68	2714.70 ± 817.92	0.65

Legend: TOLAC—trial of labor after cesarean; SD—standard deviation; CD—cesarean delivery; VD—vaginal delivery; VBAC—vaginal birth after cesarean; PE—preeclampsia; GHTN—gestational hypertension.

**Table 2:** Results from the multinomial logistic regression model that evaluated the risk of maternal and neonatal complications in cases of failed TOLAC.

Complication	Relative Risk Ratio	95%CI	*p* Value
Uterine rupture (complete/incomplete)	4.68	1.81–12.07	0.001
Anemia	1.07	0.12–9.47	0.950
Blood transfusions	1.30	0.05–3.16	0.992
ICU admission	1.79	0.70–4.58	0.03
Emergency hysterectomy	19.28	3.70–100.32	<0.001
Low Apgar score (less than 7)	2.94	1.35–6.38	0.006
Non-invasive ventilation	1.41	0.52–3.76	0.492
NICU admission	5.10	2.32–11.21	<0.001
Invasive ventilation	4.11	1.360–12.46	0.012
Intraventricular hemorrhage	1.20	0.24–5.82	0.820

Legend: CI—confidence interval; ICU—intensive care unit admission; NICU—neonatal intensive care unit admission.

**Table 3:** Probabilities of maternal and neonatal complications in cases of failed or successful TOLAC.

Complication	Probability in Case of Failed TOLAC	Probability in Case of Successful TOLAC	*p* Value
Uterine rupture (complete/incomplete)	0.26	0.07	<0.001
Anemia	0.03	0.02	0.31
Blood transfusions	0.04	0.02	0.48
ICU admission	0.11	0.03	0.003
Emergency hysterectomy	0.17	0.01	<0.001
Low Apgar score (less than 7)	0.41	0.19	<0.001
Non-invasive ventilation	0.17	0.13	0.42
NICU admission	0.47	0.14	<0.001
Invasive ventilation	0.17	0.04	0.002
Intraventricular hemorrhage	0.05	0.04	0.145

Legend: TOLAC—trial of labor after cesarean; ICU—intensive care unit admission; NICU—neonatal intensive care unit admission.

**Table 4:** Comparison of the odds of the most important adverse maternal outcomes between the evaluated groups, controlled for confounding variables.

Adverse Pregnancy Outcomes	Failed TOLAC versus Successful TOLAC							
	Crude Estimates	*p* Value	M-H Combined	*p* Value *	Magnitude of the Confounding	M-H all Predictors	*p* Value **	Magnitude of the Confounding
	OR and 95%CI		aOR and 95%CI			aOR and 95%CI		
UR ^ Maternal age	4.68 (1.76–12.43)	0.0006	4.54 (1.74–11.83)	0.0007	0.03	4.85 (0.95–24.59)	0.03	−0.04
UR ^ Obesity			4.89 (1.81–13.19)	0.0005	−0.04			
UR ^ Parity			4.65 (1.73–12.49)	0.0008	0.01			
UR ^ Previous CD			4.32 (1.64–11.39)	0.0012	0.08			
UR ^ Previous VD			4.30 (1.44–11.58)	0.0012	0.09			
UR ^ Previous VBAC			4.54 (1.70–12.06)	0.0009	0.03			
UR ^ Induced labor			5.32 (1.86–15.19)	0.0005	−0.12			
UR ^ Birthweight			4.55 (1.73–11.96)	0.0007	0.03			
UR ^ GA at birth			4.79 (1.73–12.07)	0.0007	−0.02			
EHT ^ Maternal age	19.28 (3.37–110.3)	<0.001	17.87 (3.36–95.04)	<0.001	0.08	12 (0.63–126.97)	0.03	0.61
EHT *^ Obesity			18.53 (3.24–105.9)	<0.001	0.04			
EHT^ Parity			19.13 (2.96–123.6)	<0.001	0.01			
EHT ^ Previous CD			24.80 (3.52–174.6)	<0.001	−0.22			
EHT ^ Previous VD			13.16 (2.6- 64.50)	<0.001	0.47			
EHT ^ Previous VBAC			18.75 (3.27–107.2)	<0.001	0.03			
EHT ^ Induced labor			25.12 (3.40–185.4)	<0.001	−0.23			
EHT * Birthweight			17.63 (3.29–94.35)	<0.001	0.09			
EHT ^ GA at birth			20.84 (2.98–145.8)	<0.001	−0.07			
ICU ^ Maternal age	4.31 (1.25–14.81)	0.01	4.29 (1.25–14.70)	0.01	0.00	12 (0.68–95.39)	0.01	−0.64
ICU * Obesity			4.85 (1.35–17.39)	0.01	−0.11			
ICU ^ Parity			5.02 (1.38–18.30)	0.006	−0.14			
ICU ^ Previous CD			4.46 (1.27–15.69)	0.01	−0.03			
ICU ^ Previous VD			4.67 (1.34–16.27)	0.007	−0.08			
ICU ^ Previous VBAC			4.18 (1.21–14.38)	0.01	0.03			
ICU ^ Induced labor			5.44 (1.39–21.22)	0.006	−0.21			
ICU ^ Birthweight			4.05 (1.25–13.08)	0.01	0.06			
ICU ^ GA at birth			4.74 (1.22–18.41	0.01	−0.09			

Legend: TOLAC—trial of labor after cesarean, aOR—adjusted odds ratio, CI—confidence interval, M-H—Mantel–Haenszel, UR—uterine rupture (complete or incomplete), EHT—emergency hysterectomy, ICU—maternal admission to the intensive care unit, CD—cesarean delivery, VD—vaginal delivery, VBAC—vaginal delivery after cesarean, GA—gestational age, ^—interaction between outcome and confounding variable, *—*p* value corresponding to the Mantel–Haenszel analysis considering individual confounders, **—*p* value corresponding to the Mantel–Haenszel analysis considering all confounders.

**Table 5:** Comparison of the odds of the most important adverse neonatal outcomes between the evaluated groups, controlled for confounding variables.

Adverse Pregnancy Outcomes	Failed TOLAC versus Successful TOLAC							
	Crude Estimates	*p* Value	M-H Combined	*p* Value *	Magnitude of the Confounding	M-H all Predictors	*p* Value **	Magnitude of the Confounding
	OR and 95%CI		aOR and 95%CI			aOR and 95%CI		
Low Apgar score (less than 7) ^ Birthweight	2.29 (1.02–5.11)	0.03	3.82 (1.37–10.63)	0.005	−0.40	6.48 (1.77–23.61)	0.001	−0.65
Low Apgar score (less than 7) ^ GA at birth			6.96 (1.80–26.83)	0.001	−0.67			
NICU admission ^ Birthweight	5.10 (2.23–11.65)	<0.001	16.22 (4.87–141.1)	<0.001	−0.69	18 (3.68–113.52)	<0.001	−0.72
NICU admission ^ GA at birth			12.92 (3.84–43.48)	<0.001	−0.61			
Invasive ventilation ^ Birthweight	3.31 (1.02–10.75)	0.03	4.27 (1.22–14.91)	0.01	−0.22	4.98 (1.34–18.44)	0.007	−0.34
Invasive ventilation ^ GA at birth			4.03 (1.18–13.72)	0.01	−0.18			

Legend: TOLAC—trial of labor after cesarean, aOR—adjusted odds ratio, CI—confidence interval, M-H—Mantel–Haenszel, NICU—neonatal intensive care unit, GA—gestational age, ^—interaction between outcome and confounding variable, *—*p* value corresponding to the Mantel–Haenszel analysis considering individual confounders, **—*p* value corresponding to the Mantel–Haenszel analysis considering all confounders.

## Data Availability

The datasets are available from the corresponding authors upon a reasonable request.
